# Is apomixis occurring in walnut (*Juglans regia* L.)? New data from progeny molecular tests and cytological investigations shed light on its reproductive system

**DOI:** 10.3389/fpls.2023.1270381

**Published:** 2023-12-01

**Authors:** Sahar Solhjoo, Reza Fatahi, Zabihollah Zamani, Abdolkarim Chehregani Rad, Fabio Palumbo, Gianni Barcaccia

**Affiliations:** ^1^ Department of Horticultural Sciences, Faculty of Agriculture, University of Tehran, Karaj, Iran; ^2^ Laboratory of Plant Cell Developmental Biology, Department of Biology, Bu-Ali Sina University, Hamedan, Iran; ^3^ Laboratory of Genomics for Plant Breeding, Department of Agronomy, Food, Natural Resources, Animals and the Environment (DAFNAE), University of Padova, Legnaro, Italy

**Keywords:** cyto-histology, genotyping, plant reproduction, offspring, outcrossing

## Abstract

**Introduction:**

Persian walnut (*Juglans regia*) is an economically important nut fruit species cultivated worldwide for its nutritious kernel and timber quality wood. Walnut trees are mostly hetero-dichogamous and, depending on the genotype, some cultivars are protogynous, while others are protandrous. Although selfing is possible when male and female blooms overlap, the dichogamy of the species promotes outcrossing. In addition to sexual reproduction, some reports indicate that elements of apomixis may occur in commercial orchards of walnut varieties and in the last two decades, nut production by apomixis has been reported in walnut. However, there are no reliable studies on the occurrence of apomictic reproduction based on cytoembryological observations and/or molecular marker-progeny tests. This study addresses the combined use of molecular and cytological analyses to gain new insights into the population genetics and reproduction systems of *J. regia*.

**Methods:**

We systematically analyzed the reproductive origin of individual progeny plants from 8 different cultivated walnut genotypes using microsatellite genotyping and carried out cytohistological investigations of 5 cultivated walnut genotypes arising seed sets from isolated flowers, to shed light on the mode of reproduction.

**Results and discussion:**

These cytometric and genotyping analyses did not support any asexual mode of reproduction or asexual propagation by seed and all individuals studied were identified as zygotic plants produced by crossing. Likewise, the cytological findings did not confirm completely the first component of apomixis, namely apomeiosis. On the other hand, according to histological evidence, adventitious embryony seems to take place at low frequency. Overall, our findings suggest that the occurrence of gametophytic apomixis is unlikely in *J. regia*, but sporophytic apomixis cannot be completely ruled out.

## 1 Introduction

Persian walnut (*Juglans regia*), a notable cultivated nut species of the Juglandaceae family, is a wind-pollinated deciduous tree native to Eurasia but now grown in many countries with a temperate climate (Manning, 1978). It is an economically important species cultivated worldwide for its nutritious kernel and timber quality wood. Walnut kernels generally contain approximately 60-70% fat rich in omega-3 fatty acids called alpha-linolenic acid (ALA), which makes up approximately 8-14% of the total fat content (Pereira et al., 2008).

The genus *Juglans* includes more than 20 diploid species, with 2n=2x=32 chromosomes, that are monoecious, meaning that the male and female flowers are borne separately on the same tree. In the spring, the male flowers are densely clustered on catkins hanging from the tree. Immature catkin buds first appear in leaf axils in late summer and persist over winter. During the spring, buds mature on the previous season’s shoot. Female flowers typically emerge from the terminal shoot after few leaves appear on recurrent shoots. In some cultivars, pistillate flowers can also be found at the tips of new growth lateral branches (McGranahan and Leslie, 2009).

The production of nuts depends mainly on climatic conditions, particularly during pollination, and a sufficient amount of pollen in the environment plays a crucial role in this process (Prentović et al., 2014).

It should be noted that walnuts are heterodichogamous, in which male and female flowering occur at different times. Depending on the genotype, some cultivars are protogynous, with the female blooming first, while others are protandrous, with the male blooming first. Despite the fact that walnuts are self-fertile and there is usually some overlap between male and female blooms, dichogamy promotes outcrossing (Wood, 1934; McGranahan and Leslie, 2009). In addition to sexual reproduction, evidence indicates that parthenogenetic development of walnuts has sometimes occurred in commercial orchards of single varieties located far away from other varieties after frost damage to catkins. A striking illustration of this occurred in 1929, in which freezing weather destroyed all pistils and catkins on ‘Payne’ trees. Following freezing, new pistils emerged a second time upon new growth shoots; however, catkins never developed. This second production of pistils was completely destroyed by a second freeze. The third time, pistils were produced, and some of these, without the possibility of receiving pollen, developed nuts. Despite the low percentage of seeds produced, the total number of nuts was sufficient to warrant harvesting (Wood, 1934). The primary references made in 1933-1990 suggest that apomixis may have contributed to the high and regular production of some walnut varieties originating from spring frost regions (Valdiviesso, 1990). Scanty studies have reported walnut apomictic mechanisms through cytoembryological analysis. In 1964, the origin of apomictic seeds in walnut varieties grown in Germany was attributed to adventitious embryony based on the observation that no embryo sac was evident in many ovules examined (Schanderl, 1964). These results were not confirmed by Sartorius and Stösser (1997), since no difference was observed in embryo development between open-pollinated and isolated flowers of those varieties, so it was concluded that diplospory may occur in walnut because embryo development always begins within the embryo sac by dividing of the egg cell.

In the last two decades, apomixis has been reported in walnut in several countries around the world, with bagging isolation being the method most commonly employed for investigating apomixis in walnut (**Table 1**, for details see cited references). In addition to the bagging isolation of pistillate flowers methods, Grouh et al. (2011) reported the first successful production of haploids in Persian walnut through parthenogenesis induced by gamma-irradiated (600 Gy) pollen; they evaluated 64 plants for ploidy level, and found that 59 plants (92.18%) were diploid and five plants (7.82%) were haploid.

**Table 1:** Available data on the observation of apomixis in *Juglans regia* along with information on the investigation tools.

Sources of walnut analyzed	Method of apomixis determination	Degree of apomixis reported	References
**Ten Chinese varieties**	Female flowers bagging isolation	9.5 - 15.6%	Zhang et al. (2000)
**Byelorussian and Ukrainian var. Racemosa DC**	Emasculation/spatial isolation/observation after frost-injured catkins	78.8 - 81.2%	Loiko (1990)
**Portuguese cv. Rego**	Female flowers bagging isolation/brief embryological analysis	4.6- 7%	Valdiviesso (1989).
**German cv. Esterhazy II and N.26**	Cyto-embryological analysis	–	Sartorius and Stösser (1997)
**Hungarian cv. Milotai 4/R**	Female flowers bagging isolation	58%	Asadian (2005)
**Four Chinese cultivars**	Female flowers bagging isolation/Isoenzyme analysis	13.1 - 78.6%	Peng-fei et al. (2006)
**Ten Turkish genotypes**	Female flowers bagging isolation/Mentor pollen isolating	0.5 - 6.4%	Şan and Dumanoğlu (2006)
**Chinese cv. Qinquan1**	Female flowers bagging isolation	24.7%	Guoliang et al. (2010)
**Twelve Romanian cultivars**	Female flowers bagging isolation	7.86 - 12.46%	Cosmulescu et al. (2012)
**Thirty Iranian genotypes**	Female flower bagging isolation	5- 25.75%	Sallom et al. (2023)

According to results collected in 1920-1929 in California, different Persian walnut varieties artificially pollinated by their own and other varieties of pollen, matured seeds produced either by selfing or by outcrossing (Wood, 1934). The distinction between selfed and hybrid genotypes within sexually generated plants can be achieved by performing progeny tests using molecular markers. Generally, progenies are considered maternal if their DNA fingerprints are identical to those of their seed parents, while aberrant progenies are characterized by new markers of paternal origin, and some maternal markers being absent (Barcaccia et al., 1997; Barcaccia et al., 1998). Three putative classes can be distinguished within aberrant progenies: i) BII hybrids: the fingerprint shows maternal and paternal markers, with at least one of the maternal markers being segregated; ii) BIII hybrids: all maternal markers are conserved and one or more paternal markers exist; and iii) nonhybrids: paternal markers are absent and one or more maternal markers are lacking (including selfing and/or haploid parthenogenetic plants) (Barcaccia et al., 2007). Molecular marker analyses, including Restriction Fragment Length Polymorphism (RFLP) analysis and PCR-based DNA fingerprinting and genotyping techniques, have proven to be efficient tools for the identification of maternal and aberrant individuals in different progenies of *Hypericum perforatum* (Barcaccia et al., 2007), *Citrus* (Woo et al., 2019), *Poa pratensis* (Mazzucato et al., 1995), mango (Ochoa et al., 2012), *Rosa* section *Caninae* (dogroses) (Werlemark, 2000), and *Malus sieboldii* (wild apomictic apple) (Bisognin et al., 2009). SSR and AFLP tools identified differences between the hybrids of hickory (*Carya cathayensis*) and pecan (*Carya illinoensis*) from the Juglandaceae family, such that differential bands were detected between the paternal parent and the progeny, but no differences were detected between the maternal parent and the progeny or among the progeny (Zhang et al., 2012). Moreover, molecular marker tools have been employed successfully to assess genetic diversity and structure in populations among species with different reproductive systems, such as *Lindera* species (Lauraceae) (Nakamura et al., 2021), *Taraxacum officinale* (Majeský et al., 2012), *Miconia albicans* (Dias et al., 2018), *Crataegus* species (Lo et al., 2009) and *Hypericum perforatum* (Barcaccia et al., 2006).

In facultative apomicts, where sexual and asexual reproduction occurs side by side in the same plant, each sexual event results in segregating and/or recombinant offspring (Nogler, 1984). Based on the few reports available, it is possible that progenies from walnut genotypes may be composed of asexually (genetically maternal) and sexually (genetically aberrant) derived plants. Therefore, the purposes of this study were (i) to detect and assess sexually derived seedlings produced by either selfing or outcrossing from apomictically derived seedlings using progeny tests based on molecular markers, and (ii) to investigate the main cytological steps of the female sporogenesis and gametogenesis pathway and embryo development to establish the mechanisms characterizing the reproductive biology of walnut.

In this study, we systematically analyzed the reproductive origin of individual progeny plants from 8 different cultivated walnut genotypes using molecular markers and carried out cytohistological investigations on arising seeds set by isolated flowers from 5 cultivated walnut genotypes to shed light on the mode of reproduction of *J. regia*.

## 2 Materials and methods

### 2.1 Plant material

The experiments were conducted on a walnut collection belonging to the Research Station of Department of Horticultural Sciences, University of Tehran and on walnut plants from private orchards (**Table 2**). The followings were used as plant materials for molecular progeny tests: the progeny plants of two commercial walnut genotypes (P7-M/GBP, P8-M/Pedro) whose pistils and catkins were completely destroyed by freezing event and who set seeds from the secondary blooming without developing catkins, and the progeny of six walnut genotypes (P1-M/G52_DB_41, P2-M/G44_ZT, P3-M/Jamal, P4-M/G55_PDB, P5-M/G11_1, P6-M/G51_DB) able to set seeds after destruction of catkins by frosts and whose pistillate flowers were isolated by bags (bags made of Span band polypropylene (PP) polymers).

**Table 2:** Plant material (8 maternal plus 102 progenies) of *Juglans regia* analyzed in the current study.

Population code (maternal plant)	No of progenies	Genotype/accession no.	Location	Origin
**POP1**	23	P1-M/G52_DB_41	Iran, Alborz province, Mohammad Shahr	Iran
**POP2**	27	P2-M/G44_ZT	Iran, Alborz province, Mohammad Shahr	Iran
**POP3**	3	P3-M/Jamal	Iran, Alborz province, Mohammad Shahr	Iran
**POP4**	6	P4-M/G55_PDB	Iran, Alborz province, Mohammad Shahr	Iran
**POP5**	12	P5-M/G11_1	Iran, Alborz province, Mohammad Shahr	Iran
**POP6**	17	P6-M/G51_DB	Iran, Alborz province, Mohammad Shahr	Iran
**POP7**	9	P7-M/GBP	Iran, Alborz province, Karaj	Iran
**POP8**	5	P8-M/Pedro	Iran, Alborz province, Kordan	France

Sample locations and abbreviations of maternal plants of 8 different populations.

### 2.2 Cytological analysis of ovaries and ovules by microscopy

Five walnut genotypes were used for cytohistological investigation (**Table 3**). Isolated female flowers were collected every other day up to two months after anthesis (soon after they were distinguishable from the leaves rosette), followed by sampling every four days up to one month later if sufficient seeds were available. All the collected materials were fixed in FAA solution including ethanol (96%): formaldehyde: glacial acetic acid (17:2:1) and stored in ethanol (70%), followed by dehydration in an ascendant ethanol series (80, 90, 95,100%), clearing by immersing in different proportions of absolute ethanol: toluene (2:1, 1:1, 1:2) and toluene only (repeated twice), and finally embedded in paraffin and sectioned at 7 µm slices with a rotary Micro DC 4055 microtome (Dideh Sabz, Urmia, Iran). After deparaffinization by toluene and a descendant ethanol series (100, 90, 80, 70, 50%), specimens were immersed in distilled water, stained using Meyer’s hematoxylin techniques and contrasted by eosin. Serial sections were studied under a light microscope (LABOMED model LX50 connected to LABOMED digital camera, iVu 3100) and a fluorescence microscope (BELL connected to BELL digital camera, Model BLACKL 3000, Italy). The developmental stages of the ovule, including megasporogenesis, megagametogenesis, and embryo development were evaluated from the perspective of reproductive strategy in the laboratory of Plant Cell Developmental Biology, Department of Biology, Bu-Ali Sina University, Hamedan, Iran. At least 50 flowers were analyzed for each stage listed above.

**Table 3:** Numbers of cytological stages observed in bulk ovaries (2.5- 5.5 mm in diameter) from developing fruits of five walnut (*Juglans regia*) genotypes during megasporogenesis and mega gametogenesis.

Genotype Code	Ovule No.	Megasporogenesis					Binuclear functional megaspore	Mega-gametogenesis				
		MMC	Dyad	Tetrad	FM/Dg. Tetrad	Two tetrad line		1-2n ES		4n ES	8n ES	Multiple ES
**P1-M/G52_DB-41**	123	16	14	21	10	1	2	13		9	11	5
**P2-M/G44_ZT**	102	12	17	19	12	1	0	8		12	7	4
**P4-M/G55_PDB**	25	4	0	7	7	0	0	0		2	1	0
**P5-M/G11_1**	28	4	2	5	6	0	0	2		2	2	0
**P6-M/G51_DB**	22	5	1	2	4	0	0	2		1	1	0

MMC, megasporocyte; FM, functional megaspore; Dg. Tetrad, Degenerating tetrad; ES, embryo sac; n, nuclei.

### 2.3 Genomic DNA extraction and genotyping by SSR markers

Genomic DNA was extracted from 200 mg of freeze-dried fresh leaves per sample using a modified CTAB method reported by de la Cruz et al. (1997) and Sánchez-Hernández and Gaytán-Oyarzún (2006). Thirty microsatellite regions or SSR loci of walnut were chosen from the literature (Woeste et al., 2002). The amplification efficiency and the polymorphism rate of the 30 SSR primer pairs were initially examined in singleplex and multiplex reactions on two samples chosen based on high phenotypic diversity in the laboratory of Genomics for Plant Breeding, DAFNAE, University of Padova, Italy.

The 7 SSR loci (**Table 4**) that amplified efficiently, providing unequivocal and polymorphic profiles, were then organized in two multiplexes and validated on 110 samples including the maternal parents (n=8) and their offspring (n=102; **Table 2**). The forward primers were tagged by the addition of oligo sequence tails M13, PAN1, PAN2 and PAN3 labeled to the 5’ end to be used in PCR along with the complementary fluorophore oligonucleotides 6-FAM, NED, PET and VIC. The PCR reaction consisted of diluted gDNA template (20 ng μL-1), Platinum Multiplex PCR Master Mix (1x) (Applied Biosystems, Carlsbad, CA, USA), 10% GC enhancer (Applied Biosystems), 0.05 μM tailed forward primer (Invitrogen, Carlsbad, CA, USA), 0.1 μM reverse primer (Invitrogen), 0.23 μM universal primer (Invitrogen) and sterile dd-water to reach a reaction volume of 20 μL. A 9600 Thermal Cycler (Applied Biosystems) was used with the following conditions: 5 min at 95°C, followed by 5 cycles of 95°C for 30 s, 54/58°C (T_a_ belonging to two different multiplexes) for 45 s, with the temperature being decreased by 1°C with each cycle, and 72°C for 45 s; then 35 cycles of 95°C for 30 s, 49/53°C (T_a_ belonging to two different multiplexes) for 45 s, and 72°C for 45 s, followed by the final extension step of 60°C for 30 min. Amplicons were first run by means of agarose gel electrophoresis (2% agarose): TAE buffer (1x): Sybr Safe DNA stain (1x) (Life Technologies) and visualized on an Uvidoc HD6 transilluminator (Uvitec, Cambridge, UK) equipped with a digital camera. Amplification products were then dried at 65°C for 1 h and subjected to capillary electrophoresis (ABI PRISM 3130xl Genetic Analyzer, Thermo Fisher), and the alleles were sized using Peak Scanner Software 1.0 (Applied Biosystems) with LIZ 500 (Applied Biosystems) as an internal size standard.

**Table 4:** Information on the 7 SSR loci validated using 110 walnut samples.

Locus name	Repeat motif	F Primer	R Primer	Length (bp)	Anchor	Mean T_m_ (°C)	Multiplex
**WGA 71**	(GA)6, (G)12	ACCCGAGAGATTTCTGGGAT	GGACCCAGCTCCTCTTCTCT	212	M13	56	1
**WGA 72**	(CT)14	AAACCACCTAAAACCCTGCA	ACCCATCCATGATCTTCCAA	151	PAN1	54	1
**WGA 33**	(GA)22, (GAGT)5, (GA)5	TGGTCTGCGAAGACACTGTC	GGTTCGTCGTTTGTTGACCT	230	PAN2	52	1
**WGA 65**	(CT)20	CACCGTCTTATGCCATCCTT	GTGCACTGTGGACGAAGAGA	161	M13	54	2
**WGA 24**	(T)8, (CT)18, (CT)4	TCCCCCTGAAATCTTCTCCT	TTCTCGTGGTGCTTGTTGAG	242	M13	58	2
**WGA 4**	(GT)5, (GA)15, (GA)11	TGTTGCATTGACCCACTTGT	TAAGCCAACATGGTATGCCA	241	PAN2	56	2
**WGA 42**	(GA)14	GTGGGTTCGACCGTGAAC	AACTTTGCACCACATCCACA	241	PAN3	58	2

### 2.4 Data analysis

Measures of genetic diversity, including the total number of observed alleles (N_a_), the number of effective alleles (N_e_), the observed and expected heterozygosity (Ho/He), and the Shannon index (I) of phenotypic diversity for each locus, were estimated using GenAlEx 6.5 software. The polymorphism information content (PIC) was calculated using Excel Microsatellite Toolkit as follows:


$$PIC=1-\sum_{i=1}^{n}p_{i}^{2}$$

In which *n* is the total number of alleles detected for the locus and *p_i_* is the frequency of the *i*th allele of the locus in the set of 110 samples investigated (Anderson et al., 1993).

Pairwise genetic similarity estimates were calculated between maternal plants and their progeny plants in all possible comparisons based on the Jaccard similarity index (Jaccard, 1908). Hierarchical clustering (UPGMA) with the probability value AU (approximately unbiased p value) of multiscale bootstrap resampling method (bootstrap replicates=1000) was obtained through the function implemented in the “*pvclust*” (Suzuki and Shimodaira, 2006) package of the free R software (version 4.2.2).

### 2.5 Ploidy level estimate by flow cytometry

Ploidy level was evaluated by flow cytometry analysis of stained nuclei by 4′,6-diamidino-2-phenylindole (DAPI), isolated from leaves of 40 walnut progenies in the laboratory of Genomics for Plant Breeding, DAFNAE, University of Padova, Italy. In brief, 35 progenies that -based on SSR data- were predicted to be the result of outcrossing were analyzed to identify any possible polyploid BIII hybrid. Similarly, 5 progenies that -based on SSR data - were predicted to be the result of selfing were analyzed to identify any possible parthenogenesis-derived haploid accession through flow cytometry (CyFlow Ploidy Analyzer, Sysmex, DE). Following the procedure outlined in the CyStain UV Precise protocol, Approximately, 0.5 cm^2^ of young fresh leaves were chopped with 0.5 ml of Nuclei Extraction Buffer (Sysmex Partec) and incubated for 2 minutes at room temperature. Then, each sample was filtered (30 µm CellTrics®, Sysmex) and incubated in 2 ml of staining buffer for 60 sec before analysis (blue fluorescence emission= 435-500 nm; flow rate of 4 µl/sec). Fluorescence histograms were evaluated using Flowing software 2.

## 3 Results and discussion

### 3.1 With regard to the first component of apomixis, apomeiosis, the cytohistological findings do not conform with the widely accepted concept of apomixis

We described and quantified the developmental stages of the walnut ovule in five different maternal walnut genotypes, detailing megasporogenesis and megagametogenesis to detect the predominant reproductive strategy (**Table 3**). It is necessary to specify that among the examined genotypes, micrographs were taken from two of them (i.e. P1-M/G52_DB-41 and P2-M/G44_ZT) for summarizing the main events and features of female sporogenesis and gametogenesis.

**Figure 1:**
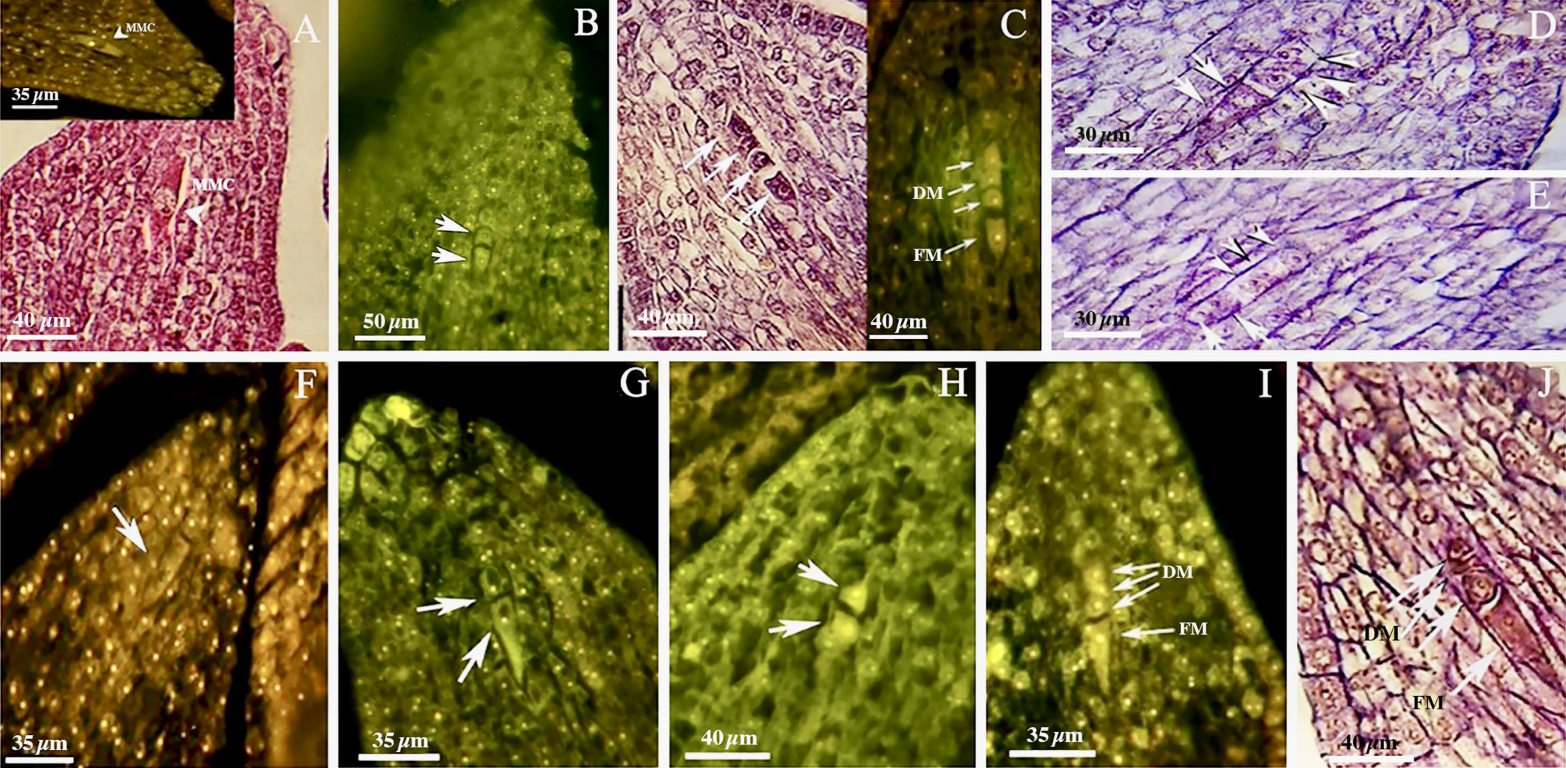
Megasporogenesis in genotype P1-M/G52_DB-41 **(A–E)** and genotype P2-M/G44_ZT **(F–J)** in longitudinal sections of the ovules. **(A)** Megasporocyte in crassinucellate ovule (arrowhead). **(B)** Cytokinesis individualizing two daughter cells in the first phase of meiosis (arrow). **(C)** Formation of a linear tetrad of megaspores (arrowhead) as result of second phase of meiosis; note that the chalazal megaspore is larger than the others. **(D, E)** Post-meiotic anomaly in megasporogenesis which binuclear functional megaspores were formed (arrow). **(F)** Megasporocyte (arrow). **(G)** Imbalance dyad (arrow). **(H)** Balance dyad (arrow). **(I, J)** Tetrad with large chalazal functional megaspore and degenerating three micropylar megaspore. MMC, Megasporocyte; FM, Functional megaspore; DM, Degenerating megaspores.

**Figure 2:**
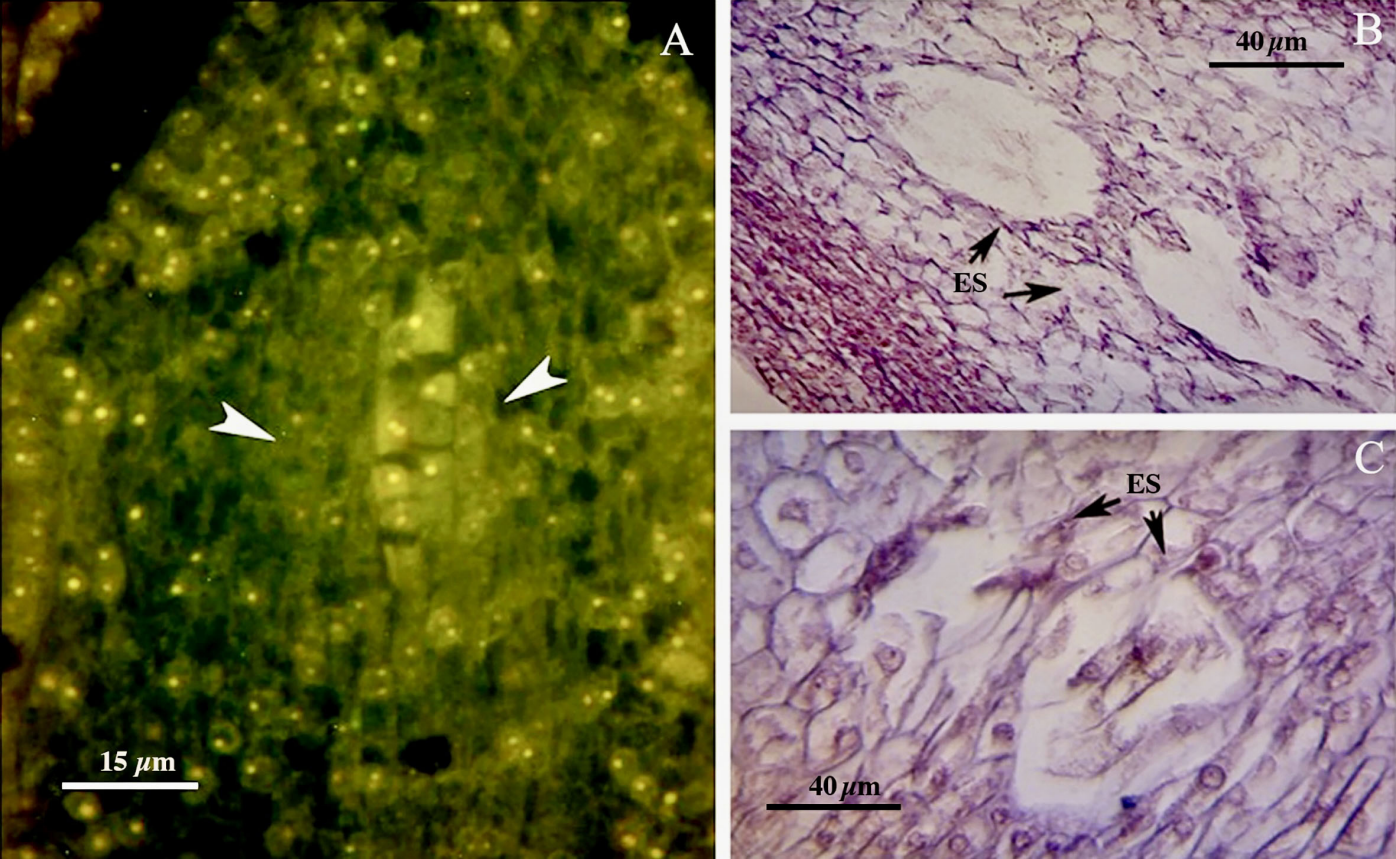
**(A)** Abnormalities including more than one linear tetrad (arrow head)**. (B, C)** Two embryo sacs lying side by side or end to end (arrows). ES, Embryo sac.

The walnut ovary was found to contain a single orthotropous ovule, which is unitegmic, crassinucellate and derived from the basal placenta (**Figure 1**). All genotypes demonstrated identical patterns during the megasporogenesis stages. A distinct megasporocyte or megaspore mother cell (MMC) was found to be situated roughly in the middle of at least five to six parietal layers that ultimately separate the MMC from the nucellar epidermis (**Figures 1A, F**). This cell progressed through the first meiosis, forming a dyad, and the second meiosis, yielding a linear tetrad of megaspores (**Figure 1**), which morphologically resembled those found in the sexual process. At this point, the functional megaspore at the chalazal end was already more extensive and more vacuolated than the others, and evolved into the embryo sac. The other three micropylar megaspores naturally degenerated (**Figures 1C, I, J**). MMCs typically underwent complete meiosis, except in some cases (0.8-1%; **Table 3**), in which abnormalities were found, including more than one linear tetrad (**Figure 2A**), followed by two embryo sacs (4%; **Table 3**) lying side by side or end to end (**Figures 2B, C**). Additionally, (2%; **Table 3**), binuclear functional megaspores (**Figures 1D, E**) were rarely observed instead of normal uninuclear products, possibly indicating post-meiotic restitution or endoreduplication.

**Figure 3:**
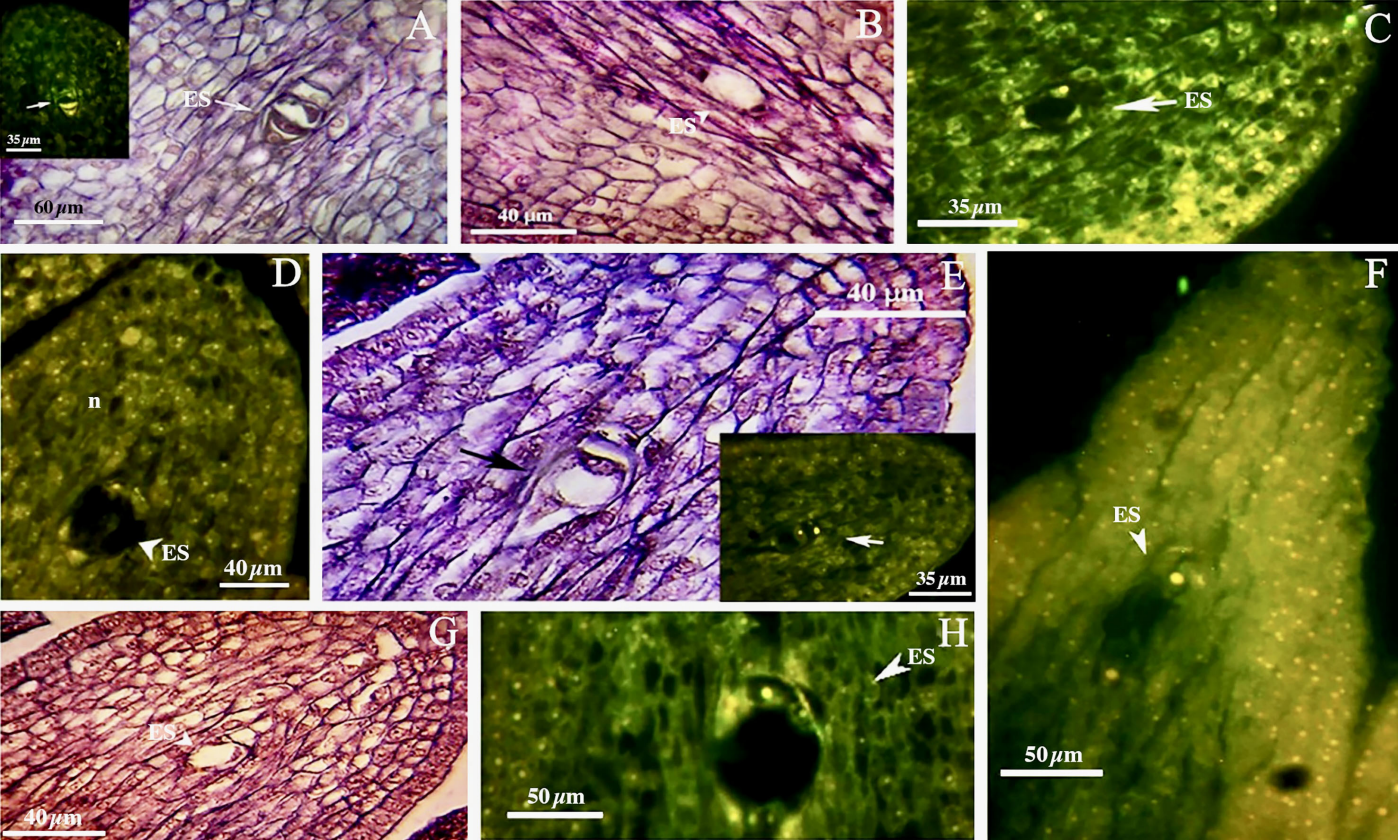
First phase of mega-gametogenesis in genotype P1-M/G52_DB-41 **(A–D)** and genotype P2-M/G44_ZT **(E–H)**, longitudinal sections of the ovules. **(A, E)** Megagametophyte development from the beginning of the first mitosis. **(B, F)** Two-nucleated megagametophyte. **(C, G)** Four-nucleated megagametophyte after the second mitosis. **(D, H)** Eight-nucleated megagametophyte after the third mitosis. ES, Embryo sac; n, Nucellus.

**Figure 4:**
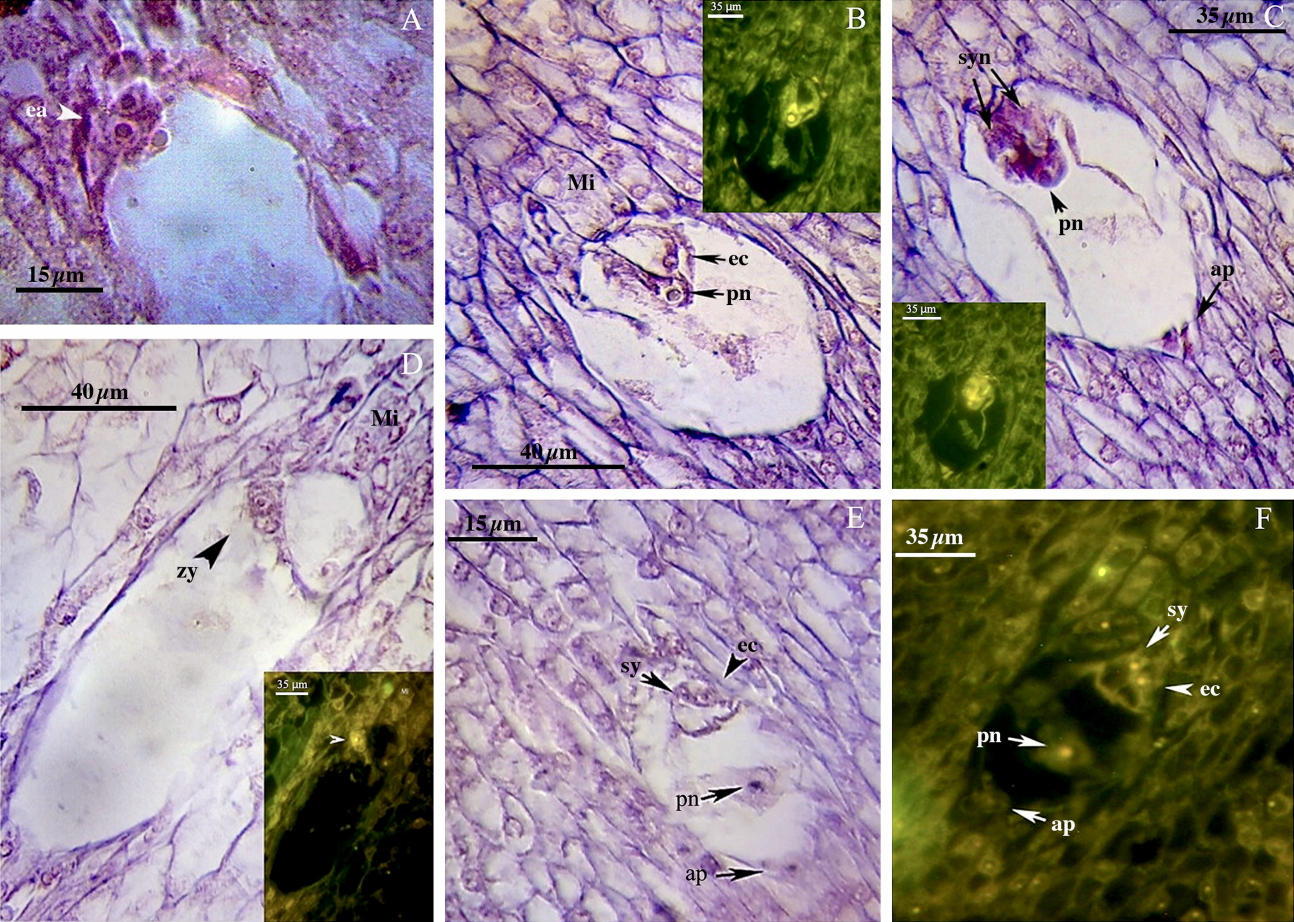
Late phase of mega-gametogenesis in P2-M/G44_ZT **(A–D)** and P1-M/G52_DB-41 **(E, F)** genotypes, longitudinal sections of embryo sac. **(A)** ea, Egg apparatus; Sy, Synergid; pn, Polar nuclei; zy, Zygote; ec, Egg cell; ap, Antipodes; Mi, Micropylar zone.

**Figure 5:**
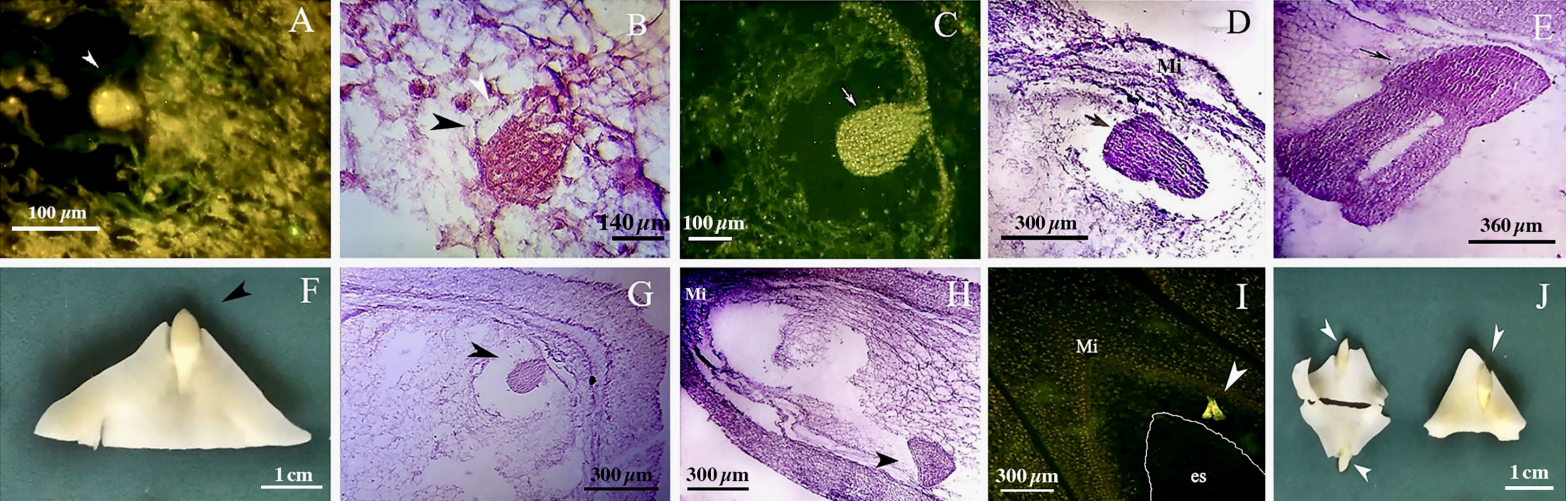
Embryo development in genotype P1-M/G52_DB-41. **(A)** 4-cell embryo. **(B)** 64-cell embryo. **(C)** globular embryo. **(D)** heart shape embryo. **(E)** cotyledon shape embryo. **(F, G)** embryo positioned at right zone close to micropylar end. **(H)** embryo positioned at wrong zone far from micropylar end. **(I)** embryo-like structure positioned outside of the embryo sac. **(J)** Polyembryony and Embryos located far from micropylar zone. Embryo, arrow and head arrow. es, embryo sac.

We found no significant differences between the genotypes during the early and final mega-gametogenesis phases. Three consecutive mitotic divisions progressed through a functional megaspore to generate an eight-nucleate embryo sac. A two-nucleate embryo sac was formed as a consequence of the first mitosis, in which two nuclei migrated to the opposite poles of the micropylar-chalazal axis (**Figures 3A, B, E, F**). These two nuclei were then divided into a four-nucleate embryo sac (**Figures 3C, G**). Eight nuclei (**Figures 3D, H**), four at the chalazal pole and four at the micropylar pole, resulted after the third and final mitosis. All genotypes demonstrated the polygonum type (seven-celled/eight-nucleate) of the embryo sac (**Table 3**), recognized by three antipodals, two polar nuclei, and an egg apparatus (two synergids and the egg cell) in the final phase of mega-gametogenesis (**Figure 4**). Both embryo and endosperm tissues followed developmental stages typical of fertilized ovaries after double fertilization. Once the embryo reached the 32 or 64-cell stage, the cellular endosperm constitution was quite evident at micropylar regions (**Figure 5B**). Further embryo development stages including, 8-cell, 64-cell, globular, heart-shaped, torpedo-shaped, and cotyledon stage (**Figures 5A–E**) conformed the zygotic nature of the embryos located in the expected region, close to micropylar end (**Figures 5F, G**). Nearly 17% of the embryos were found positioned far from the expected micropylar end zone, at the middle of embryo sac (**Figures 5H–J**; **Table 5**). Furthermore, we observed that very few ovules contained an embryo-like structure outside the embryo sac (adventitious embryony) and or polyembryony in genotype P1-M/G52_DB-41 (**Figures 5I, J**).

**Table 5:** Observed positions of embryos in ovules from walnut genotypes. following positions:.

Genotype Code	Ovule no.	Embryo no. (Positioned at micropylar end)	Embryo no. (Positioned far to micropylar end)	Embryo no. (Positioned outside the ES)
**P1-M/G52_DB-41**	46	31	8	1
**P2-M/G44_ZT**	21	2	1	0

Based on cytological data available in the literature, both diplosporous apomixis and adventitious embryony seems to be possible in *J. regia*. Despite Sartorius and Stösser (1997) hypothesized that walnut could be characterized by diplospory, our cytohistological results did not support the occurrence of apomeiosis. However, we observed few post-meiotic anomalies in megasporogenesis and, in particular, the formation of binuclear functional megaspores in genotype P1-M/G52_DB_41. This was previously observed in *Miconia albicans* after meiosis II, supporting the hypothesis of endoreduplication or restitution events (Caetano et al., 2013). However, in our genotypes the MMC typically went through complete meiosis and the ovules displayed the typical polygonum type development of the embryo sac. As a matter of fact, neither diplosporic mechanisms nor aposporic initials have been documented. Our results indicated that embryo development started with egg cell division inside the embryo sac. About 80% of embryos were positioned close to the micropylar end zone inside the embryo sac. The observation of a few embryos positioned far from micropylar end with unknown origin and one embryo outside the embryo sac were in agreement with Schanderl (1964) and Valdiviesso (1990) were the apomixis-derived embryo resulted located apart from micropylar end from nucellus somatic cells, as a result of adventitious embryony.

### 3.2 Based on microsatellite genotyping and cytometric results, asexual reproduction strategy is unlikely to occur in the studied walnut genotypes

We used 7 polymorphic SSR nuclear loci to genotype 102 progenies obtained from controlled crosses (isolated by bagging) and after a second pistillate flowers production (after freezing events). The related mother plants (n=8) were also genotyped (**Table S1**). Overall, thirty-five marker alleles, ranging from 4 (WGA 4, and WGA 42) to 7 alleles (WGA 65) per locus, were observed among the 110 walnut individuals with a mean value of 5 alleles per locus (**Table 6**). This value was in full agreement with what observed by Ebrahimi et al. (2011) (i.e. 5.1), but lower than those reported by Aradhya et al. (2010), and Ebrahimi et al. (2017) (10 and 7, respectively), possibly due to the small number of mother plants initially used in the experiment. The number of effective alleles ranged from 2.11 (WGA 42) to 5.26 (WGA 65). Based on the Botstein et al. (1980) classification, all the SSRs used were highly polymorphic, with PIC values always exceeding 0.5. The mean value of the polymorphism information content (PIC) across all marker loci was 0.61, with estimates ranging from a minimum of 0.52 (WGA 42) to a maximum of 0.81 (WGA 65). In addition, the mean value of Shannon’s information index (I) was 1.12, ranging from 0.82 (WGA 42) to 1.74 (WGA 65) (**Table 6**), lower than the values reported by Vahdati et al. (2015) (I = 1.79). The analysis of plant genotypes revealed an average observed heterozygosity (Ho) equal to 0.65, varying from 0.35 to 0.95. Overall, the average heterozygosity for single individuals was found to be relatively high, consistently with the putatively high outcrossing rates of walnut.

**Table 6:** SSR descriptive statistics reporting marker locus name, sample size of individuals successfully amplified for each locus, number of observed alleles (Na), number of effective alleles (Ne), observed heterozygosity (Ho), expected heterozygosity (He), Shannon’s information index (I) and polymorphic information content (PIC).

Marker	N	Na	Ne	Ho	He	I	PIC
**WGA 65**	110	7.00	5.26	0.95	0.81	1.74	0.81
**WGA 71**	110	5.00	2.80	0.65	0.64	1.21	0.64
**WGA 24**	110	5.00	2.25	0.35	0.56	1.06	0.61
**WGA 33**	110	5.00	2.61	0.89	0.62	1.11	0.62
**WGA 4**	110	4.00	2.27	0.74	0.56	0.93	0.56
**WGA 42**	110	4.00	2.11	0.47	0.53	0.82	0.52
**WGA 72**	110	5.00	2.13	0.45	0.53	0.98	0.53
**MEAN**	110	5.00	2.78	0.65	0.61	1.12	0.61
**SE**	0	0.38	0.42	0.09	0.04	0.11	0.04

**Figure 6:**
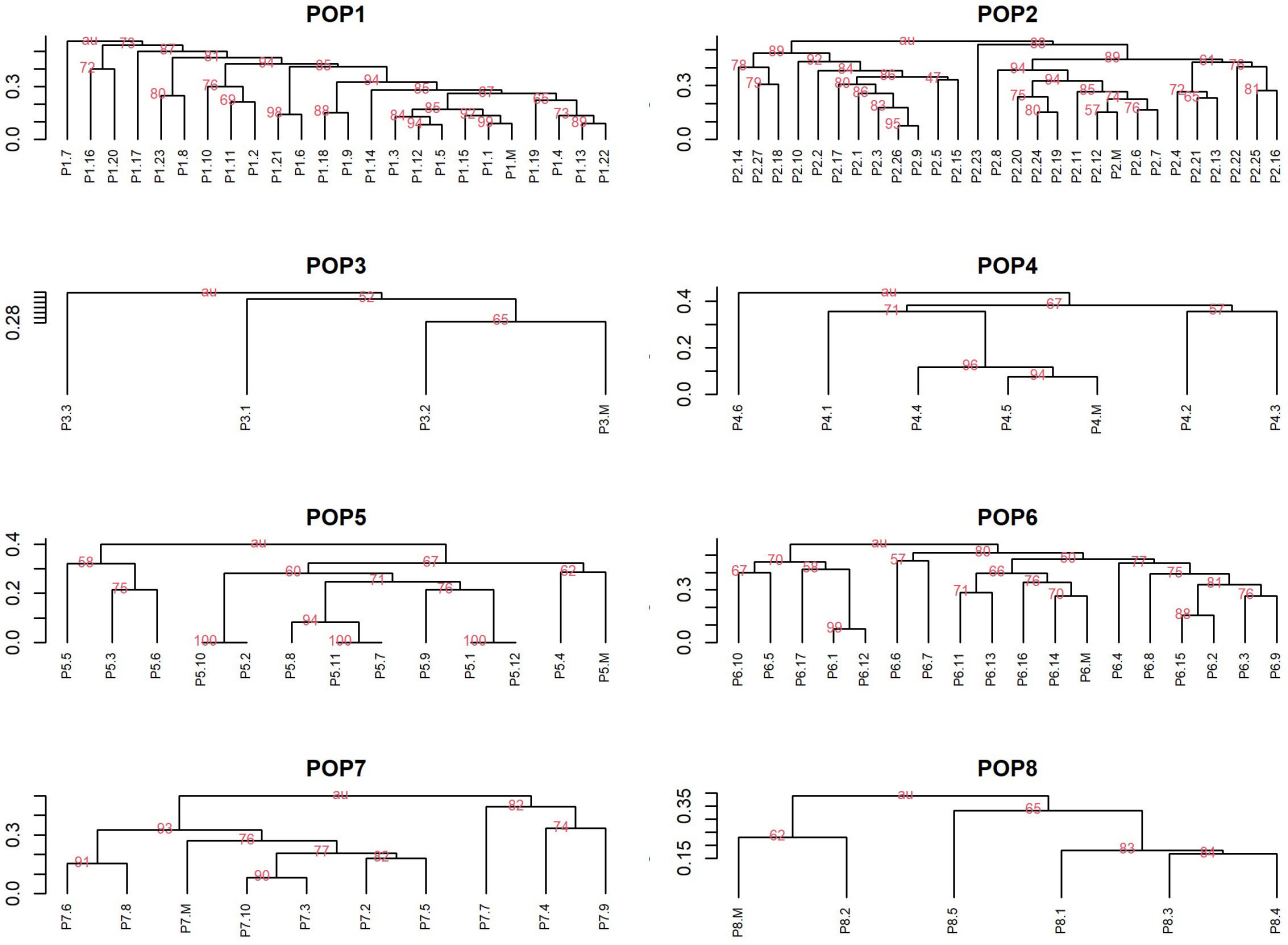
UPGMA dendrograms based on pairwise genetic similarity matrixes. Dendrograms for eight walnut population using UPGMA method based on the Jaccard similarity index showing genetic similarity within populations including progeny plants and maternal genotypes (indicated as M) of *Juglans regia*. Only bootstrap values above 50 were reported in the Figure.

**Figure 7:**
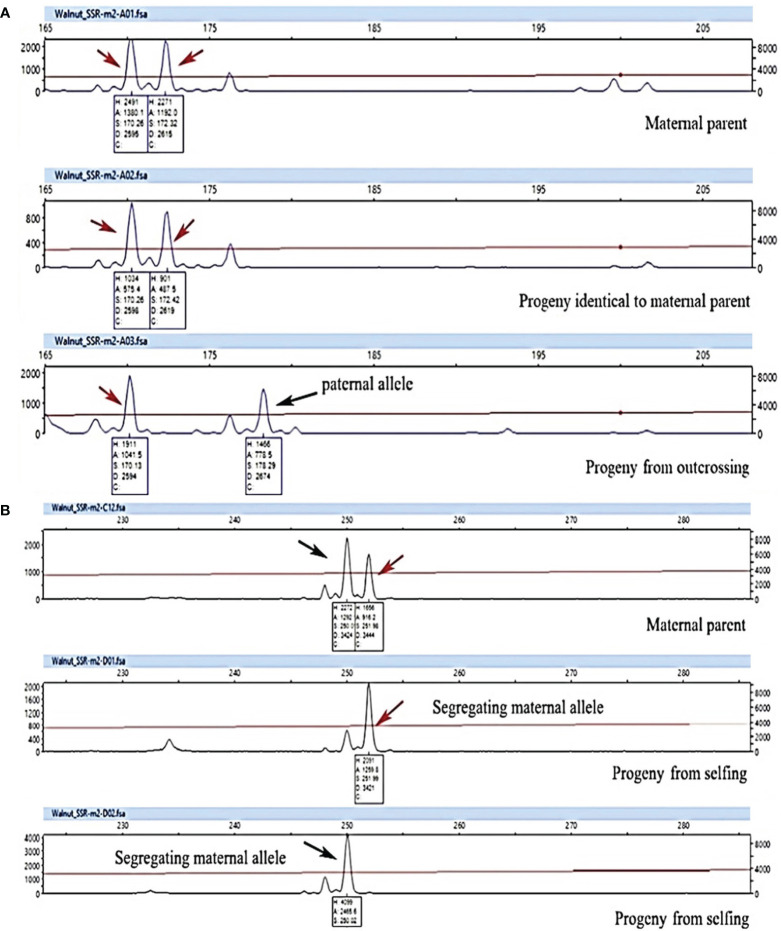
Genotyping analysis of progenies using WGA65 marker **(A)** and WGA4 marker **(B)** to detect progeny origin and reproductive strategy in 8 walnut genotypes. A plant was considered the result of selfing (non-hybrids) when paternal markers were absent and one maternal marker was lacking, whereas it was considered the result of outcrossing when displaying one maternal and one nonmaternal (i.e. paternal) markers. Finally, a progeny was considered the result of apomixis event when all maternal markers conserved. Arrows indicate allele peaks detected.

All maternal genotypes generated unique molecular profiles based on the seven marker loci (**Table S1**) and resulted therefore distinguishable from each other. The mother plants were heterozygous for at least 3 (up to 6) microsatellite loci, which was sufficient to distinguish different origins of reproduction. Each progeny plant was analyzed for all seven SSR loci and a UPGMA tree was then constructed for each population using the matrix of the genetic similarity estimates calculated in all possible pairwise combinations (**Figure 6**). Based on the genotyping data, three possible scenarios were expected: i) offspring with a molecular profile identical to that of the mother plant, likely resulting from apomixis (**Figure 7A**); ii) offspring with a molecular profile characterized by maternal and non-maternal (paternal) alleles, and therefore the result of outcrossing (**Figure 7A**); iii) offspring with a molecular profile characterized by some of the possible maternal alleles (and no non-maternal alleles), likely resulting from selfing (**Figure 7B**).

Considering all 7 SSR loci, none of the offspring was genetically identical to their mother parent and hence apomixis was not recorded in the investigated genotypes (**Figure 6** and **Table 7**).

**Table 7:** Progeny plants origin aroused from 8 walnut genotypes based on the allele pattern at selected SSR loci.

Progeny no.	Similarity Coefficient ^1^	Progeny Origin		Progeny no.	Similarity Coefficient	Progeny Origin	
		Sexuality				Sexuality	
		Selfing	Outcrossing			Selfing	Outcrossing
**P1-M**		4	19	**P3- 3**	0.86		•
**P1- 1**	0.97	•		**P4-M**		2	4
**P1- 2**	0.91		•	**P4- 1**	0.83		•
**P1- 3**	0.97	•		**P4- 2**	0.86		•
**P1- 4**	0.97	•		**P4- 3**	0.88		•
**P1- 5**	0.97		•	**P4- 4**	0.94	•	
**P1- 6**	0.83		•	**P4- 5**	0.97	•	
**P1- 7**	0.77		•	**P4- 6**	0.82		•
**P1- 8**	0.88		•	**P5-M**		0	12
**P1- 9**	0.86		•	**P5- 1**	0.86		•
**P1-10**	0.83		•	**P5- 2**	0.86		•
**P1-11**	0.83		•	**P5- 3**	0.83		•
**P1-12**	0.94		•	**P5- 4**	0.88		•
**P1- 13**	0.91		•	**P5- 5**	0.74		•
**P1- 14**	0.91		•	**P5- 6**	0.74		•
**P1- 15**	0.97	•		**P5- 7**	0.88		•
**P1- 16**	0.8		•	**P5- 8**	0.91		•
**P1- 17**	0.83		•	**P5- 9**	0.88		•
**P1- 18**	0.86		•	**P5- 10**	0.86		•
**P1- 19**	0.88		•	**P5- 11**	0.88		•
**P1- 20**	0.74		•	**P5- 12**	0.86		•
**P1- 21**	0.83		•	**P6-M**		0	17
**P1- 22**	0.94		•	**P6- 1**	0.71		•
**P1- 23**	0.8		•	**P6- 2**	0.83		•
**P2- M**		0	27	**P6- 3**	0.83		•
**P2- 1**	0.8		•	**P6- 4**	0.74		•
**P2- 2**	0.8		•	**P6- 5**	0.8		•
**P2- 3**	0.77		•	**P6- 6**	0.77		•
**P2- 4**	0.83		•	**P6- 7**	0.8		•
**P2- 5**	0.8		•	**P6- 8**	0.86		•
**P2- 6**	0.94		•	**P6- 9**	0.88		•
**P2- 7**	0.94		•	**P6- 10**	0.8		•
**P2- 8**	0.86		•	**P6- 11**	0.83		•
**P2- 9**	0.77		•	**P6- 12**	0.74		•
**P2- 10**	0.74		•	**P6- 13**	0.83		•
**P2- 11**	0.94		•	**P6- 14**	0.88		•
**P2- 12**	0.94		•	**P6- 15**	0.88		•
**P2- 13**	0.77		•	**P6- 16**	0.88		•
**P2- 14**	0.8		•	**P6- 17**	0.8		•
**P2- 15**	0.77		•	**P7-M**		0	9
**P2- 16**	0.89		•	**P7- 1**	0.88		•
**P2- 17**	0.74		•	**P7- 2**	0.91		•
**P2- 18**	0.8		•	**P7- 3**	0.91		•
**P2- 19**	0.91		•	**P7- 4**	0.83		•
**P2- 20**	0.86		•	**P7- 5**	0.91		•
**P2- 21**	0.86		•	**P7- 6**	0.83		•
**P2- 22**	0.8		•	**P7- 7**	0.8		•
**P2- 23**	0.77		•	**P7- 8**	0.83		•
**P2- 24**	0.91		•	**P7- 9**	0.77		•
**P2- 25**	0.86		•	**P8-M**		0	5
**P2- 26**	0.8		•	**P8- 1**	0.8		•
**P2- 27**	0.74		•	**P8- 2**	0.91		•
**P3-M**		1	2	**P8- 3**	0.86		•
**P3- 1**	0.86		•	**P8- 4**	0.8		•
**P3- 2**	0.88	•		**P8- 5**	0.8		•

M indicates the mother plant of each progeny.

^1^Similarity coefficient between progeny and its maternal plant.

**Figure 8:**
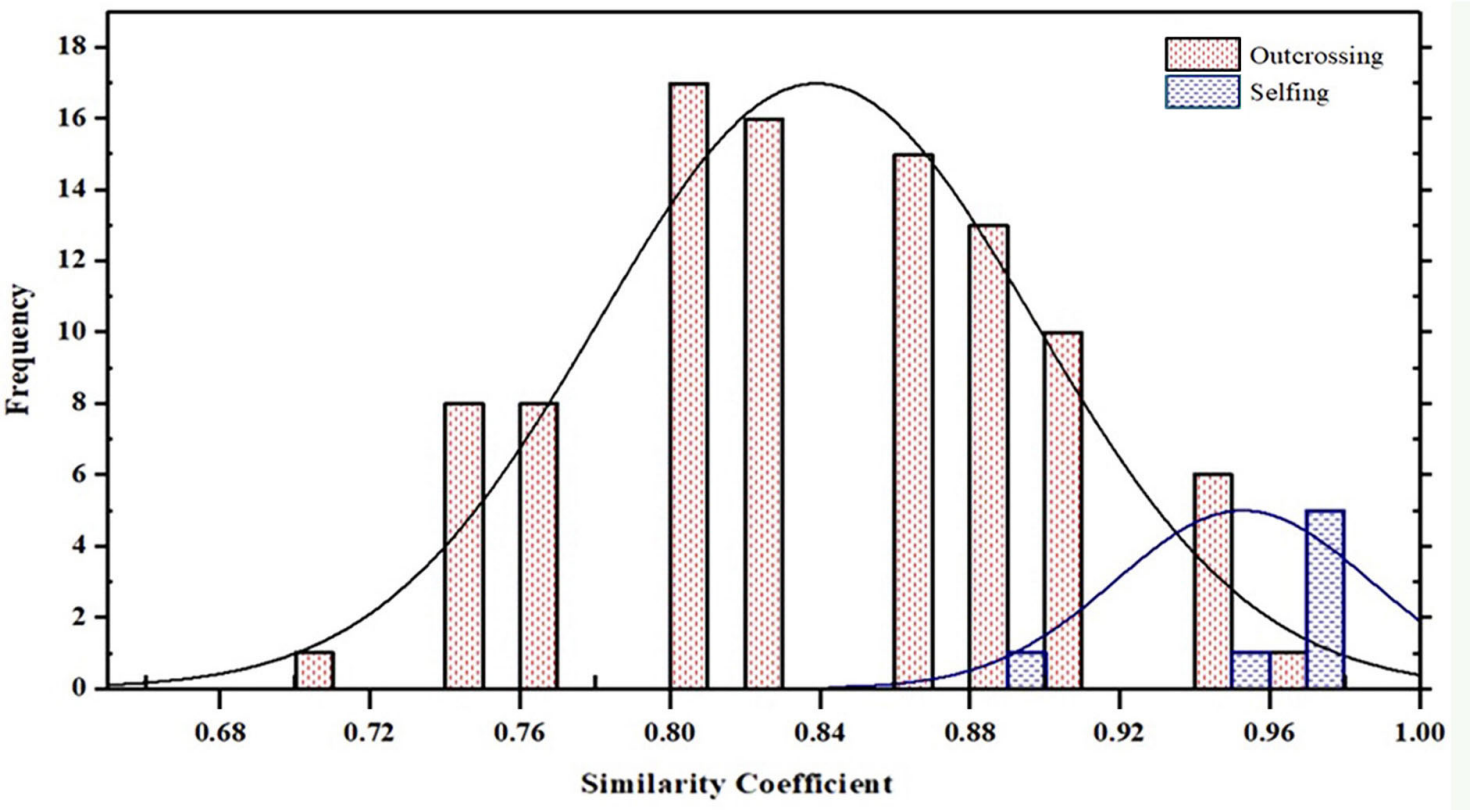
Frequency histogram of similarity coefficient between progeny originated by outcrossing and selfing and their mother parent among 8 populations in *Juglans regia*.

Populations 2, 5, 6, 7 and 8 resulted exclusively constituted by progenies deriving from outcrossing. Indeed, each of the above-mentioned offspring showed non-maternal alleles in at least one SSR locus. In contrast, the proportion of progenies originated by selfing in populations 1, 3 and 4 ranged between 17% and 33% (**Table 7**). The similarity coefficient of individual progenies originated by outcrossing when compared to their mother parent ranged from a minimum of 0.71 to a maximum of 0.95. By contrast, the genetic similarity values calculated between progenies originated by selfing and their mother parent ranged from 0.88 to 0.97 (**Table 7** and **Figure 8**).

**Figure 9:**
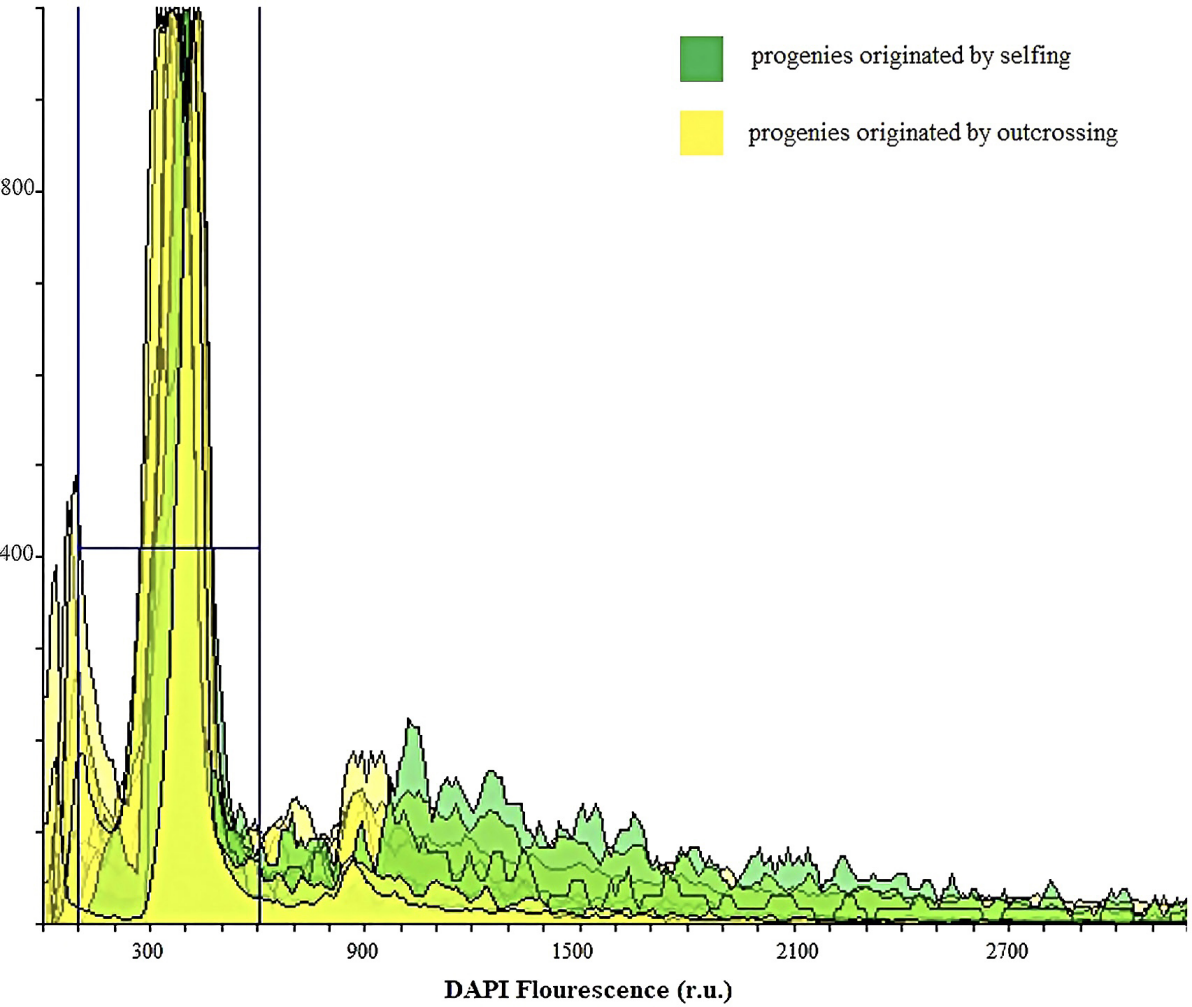
Ploidy evaluation by flow cytometry analysis of DAPI stained nuclei in suspensions isolated from leaves of 40 walnut progenies to identify BIII hybrids and progeny originated by selfing from haploid parthenogenesis: The peaks in green indicate the diploid progenies originated by selfing, whereas the yellow-colored peaks represent diploid progenies originated by outcrossing.

All the samples (n=40) analyzed by flow cytometry resulted diploids (**Figure 9**). For 33 out of 40 samples, this finding was in agreement with the SSR data. On the contrary, the remaining seven samples were expected to be triploids, in light of the WGA 65 triallelic SSR locus. This result partially confirms the hypothesis according to which a single three-allelic locus does not always imply triploidy (Draga et al., 2023). Furthermore, since no haploid or polyploid sample was observed, the cytometric analysis was also useful to demonstrate the total lack of accessions classifiable as non-hybrid or BIII hybrid.

Apomixis rates (defined as fruit set rate after bagging isolation) of 9.5-15.6% (Zhang et al., 2000), 13.1-76.8% (Guo-Liang et al., 2007) and 24.7% (Guoliang et al., 2010) have been reported for walnut trees grown in China. On the other hand, in the Romanian and Turkish walnut cultivars studied by Cosmulescu et al. (2012) and Şan and Dumanoğlu (2006), data showed that the seed set rate after bagging was low and insufficient for commercial seed and crop production. Bagging isolation has been so far the most commonly employed method for investigating apomixis in walnut. However, in most of the previous studies, the twigs containing flowers were typically protected by a single bag and, in species were the pollen is mostly wind dispersed (including walnut), the risk of contamination by pollen remains very high (Saomitou-Laprade et al., 2017). In this study, despite isolating female flowers by bagging and considering that the male catkins of the genotypes studied were mainly destroyed by the spring frost, we proved that 92% of progeny plants were derived from outcrossing, and 8% were produced by selfing. This would suggest that bagging isolation alone cannot be relied upon to determine whether apomixis occurs in walnut, and that complementary molecular progeny tests and cytological analyses of the progenies are needed. Our genotyping analyses did not support any asexual mode of reproduction and all individuals studied were identified as zygotic plants. Based on cytometric analysis, all progenies resulting from outcrossing and selfing were diploids, indicating that triploid and haploid status was not prevalent among the tested genotypes. On the other hand, based on cytohistological results, gametophytic apomixis is unlikely to occur in the walnut genotypes studied, although adventitious embryony cannot be completely ruled out, as the embryos derived from sporophytic apomixis are commonly diploid.

The present findings resulted in agreement with general expectations considering previous reports in walnut (Wood, 1934; Loiko, 1990). According to these studies, some pistils are produced at second or third time upon new flash growth following the freeze event, apparently without any possible chance of receiving pollen or developing nuts. In the absence of further complementary evidence, it has so far been hypothesized that a possible progeny may have been formed via apomixis (even with rates up to 78.8% - 81.2%). On the contrary, combining cytohistological, genotypic and flow citometry analyses, we were able to prove that all the progenies originated through a sexual process and with a high outcrossing rate, despite the use of bagged mother plants whose primary pistils and catkins were completely destroyed by freezing weather, and whose secondary blooming did not involve catkins development.

## 4 Conclusion

Although the possibility of apomictic seed set based on female flower bagging isolation and few brief histological studies have been reported in *J. regia*, our flow cytometric and genotyping analyses did not support any asexual mode of reproduction and all individuals studied were identified as zygotic plants. Likewise, our cytological findings showed that mega-gametogenesis produced a polygonum-type embryo sac in all genotypes and the embryos developed correctly through fertilization of the egg cell. Therefore we did not confirm the first component of apomixis (apomeiosis) in walnut. In contrast, our cytohistological studies indicated that adventitious embryony occurs at a very low frequency in these genotypes. The occurrence of gametophytic apomixis is therefore unlikely in *J. regia*, but sporophytic apomixis cannot be completely ruled out in this species. However, the genotyping results confirmed that the walnut seeds produced after freezing events from the second and third pistillate flowers production and with no staminate flowers available, all resulted from sexual reproduction, mainly from outcrossing events.

## Data availability statement

The original contributions presented in the study are included in the article/**Supplementary Material**. Further inquiries can be directed to the corresponding author.

## Author contributions

SS: Conceptualization, Formal Analysis, Investigation, Methodology, Writing – original draft. RF: Conceptualization, Funding acquisition, Project administration, Supervision, Writing – review & editing. ZZ: Conceptualization, Supervision, Writing – review & editing. ACR: Funding acquisition, Methodology, Supervision, Writing – review & editing. FP: Supervision, Writing – review & editing. GB: Conceptualization, Funding acquisition, Methodology, Supervision, Writing – review & editing.
