# Supplementary material for: Is apomixis occurring in walnut (Juglans regia L.)? New data from progeny molecular tests and cytological investigations shed light on its reproductive system
Source: Front Plant Sci. 2023 Dec 1;14:1270381. doi: 10.3389/fpls.2023.1270381 (PMC10792767; doi:10.3389/fpls.2023.1270381)
Supplement: Supplementary file 1 [file Table_1.docx]

***Supplementary Material***

**Is apomixis occurring in walnut (*Juglans regia* L.)? New data from progeny molecular tests and cytological investigations shed light on its reproductive system**

Sahar Solhjoo, Reza Fatahi^*^, Zabihollah Zamani, Abdolkarim Chehregani Rad, Fabio Palumbo and Gianni Barcaccia^*^

*** Correspondence:**

Reza Fatahi: [fattahi@ut.ac.ir](mailto:fattahi@ut.ac.ir) ,

Gianni Barcaccia: [gianni.barcaccia@unipd.it](mailto:gianni.barcaccia@unipd.it)

Table S1: Genotypes at 7 SSR loci of the maternal lines and their progeny used in 8 populations of *Juglans regia*

| Maternal line code | Population No. | SSR Loci |
| --- | --- | --- |
|  |  | **WGA65 WGA71 WGA24 WGA33 WGA04 WGA42 WGA72** |
| P1-M/ G52_DB_41 | **1** | 164:170:172 224:225 296:296 213:221 250:252 227:227 164:164 |
| P2-M/ G44_ZT | **2** | 172:174 224:224 281:281 213:213 250:260 227:231 164:164 |
| P3-M/ Jamal | **3** | 164:176 224:224 281:283 213:220 252:260 227:231 168:170 |
| P4-M/G55_PDB | **4** | 172:178 224:226 258:281 213:220 250:252 227:227 162:164 |
| P5-M/ G11_1 | **5** | 164:176 224:226 281:281 213:220 250:252 227:227 162:170 |
| P6-M/G51_DB | **6** | 174:178 224:226 283:296 213:220 250:252 231:231 162:168 |
| P7-M/GBP | **7** | 174:176 222:226 281:281 213:220 250:250 231:231 164:164 |
| P8-M/Pedro | **8** | 164:174 224:226 254:281 213:221 250:252 231:231 164:164 |
| Progeny No. | **Population No.** | **WGA65 WGA71 WGA24 WGA33 WGA04 WGA42 WGA72** |
| 1 | **1** | 164:170:172 224:225 296:296 213:213 250:252 227:227 164:164 |
| 2 | **1** | 170:**178** 224:225 **281**:296 213:221 250:252 227:227 164:164 |
| 3 | **1** | 170:172 224:224 296:296 213:221 250:252 227:227 164:164 |
| 4 | **1** | 172:172 224:225 296:296 213:221 250:252 227:227 164:164 |
| 5 | **1** | 170:172 224:225 **281**:296 213:221 250:252 227:227 164:164 |
| 6 | **1** | 170:172:**176** **222**:224 **281**:296 **220**:221 250:252 227:**231** 164:164 |
| 7 | **1** | 164:170:**180** 224:224 **281**:296 213:**220** 252:252 227:**233** 164:164 |
| 8 | **1** | 170:172 225:225 **281**:296 221:**225** 250:252 227:227 164:164 |
| 9 | **1** | **164**:172:**176** **222**:224 **281**:296 213:221 252:252 227:227 164:164 |
| 10 | **1** | 170:**178** 224:225 296:296 213:**225** 250:252 227:**231** **162**:164 |
| 11 | **1** | 172:**176**:**178** 224:225 **281**:296 221:**223** 250:252 227:**231** 164:164 |
| 12 | **1** | 170:172 224:224 **281**:296 213:**220** 250:252 227:227 164:164 |
| 13 | **1** | **164**:172 224:224 296:296 213:221 250:252 227:227 164:164 |
| 14 | **1** | **164**:170 224:224 296:296 213:221 250:252 227:227 164:164 |
| 15 | **1** | 170:172 225:**225** 296:296 213:221 250:252 227:227 164:164 |
| 16 | **1** | **164**:170 225:**226** 296:296 213:221 250:250 227:**231** **162**:164 |
| 17 | **1** | 170:172 **222**:224 258:296 213:221 252:**254** 227:**233** 164:164 |
| 18 | **1** | **164**:172 **222**:225 **281**:296 213:221 250:252 227:227 164:164 |
| 19 | **1** | **164**:172 224:225 296:296 213:**220** 250:252 227:227 164:164 |
| 20 | **1** | **164**:172 225:**226** **281**:296 213:**220** 250:250 227:227 **162**:164 |
| 21 | **1** | 170:172 **222**:224 **281**:296 213:**220** 250:252 227:**231** 164:164 |
| 22 | **1** | **164**:172 224:225 296:296 213:221 250:252 227:227 164:164 |
| 23 | **1** | **164**:170 225:225 **281**:296 221:**225** 250:250 227:227 164:164 |
| 1 | **2** | **164**:174 224:**226** 281:281 213:**220** 250:250 227:227 164:164 |
| 2 | **2** | **164**:172 224:**226** 281:281 213:213 250:250 231:231 164:**166** |
| 3 | **2** | **164**:172 224:**226** **258**:281 213:**220** 250:260 227:227 **162**:164 |
| 4 | **2** | **170**:172 224:224 281:281 213:**225** 250:**252** 227:231 164:164 |
| 5 | **2** | 174:**176** 224:**226** 281:281 213:**220** 250:260 231:231 **162**:164 |
| 6 | **2** | 172:174 224:**225** 281:281 213:**221** 250:250 227:231 164:164 |
| 7 | **2** | 172:174 224:224 281:281 213:**221** 250:**252** 227:231 164:164 |
| 8 | **2** | **170**:174 **222**:224 281:281 213:213 **252**:260 227:231 164:**168** |
| 9 | **2** | **164**:174:**176** 224:**226** **258**:281 213:**220** 250:250 227:231 **162**:164 |
| 10 | **2** | 172:**178** 224:**226** **258**:281 213:**220** 250:250 227:227 **162**:164 |
| 11 | **2** | 172:174 **222**:224 281:281 213:**221** 250:260 227:231 164:164 |
| 12 | **2** | 172:174 **222**:224 281:281 213:**221** 250:260 227:231 **162**:164 |
| 13 | **2** | **164**:172 **222**:224 **254**:281 213:**225** 250:**252** 227:231 164:164 |
| 14 | **2** | **164**:174 **222**:224 281:281 213:**220** **252**:260 227:231 164:164 |
| 15 | **2** | **164**:172 224:**226** **258**:281 213:**220** 250:260 231:231 **162**:164 |
| 16 | **2** | **164**:172 224:224 281:281 213:213 250:260 231:231 164:164 |
| 17 | **2** | **164**:174 224:**226** 281:281 213:**220** 250:250 231:231 **162**:164 |
| 18 | **2** | 174:174 **222**:224 281:281 213:**220** 250:**252** 231:231 164:164 |
| 19 | **2** | **170**:174 224:**225** 281:281 213:**221** 250:260 227:231 164:164 |
| 20 | **2** | **170**:172 224:**225** 281:281 213:213 250:250 227:231 164:164 |
| 21 | **2** | **164**:172 224:224 281:281 213:213 250:**252** 227:231 164:164 |
| 22 | **2** | **164**:172 **222**:224 281:281 213:**221** 250:250 231:231 **162**:164 |
| 23 | **2** | **164**:172 **222**:224 281:281 213:**225** 250:260 231:231 164:**166** |
| 24 | **2** | **170**:172 224:**225** 281:281 213:**221** 250:260 227:227 164:164 |
| 25 | **2** | **164**:172 224:224 281:281 213:**221** **252**:260 231:231 164:164 |
| 26 | **2** | **164**:174 224:**226** **258**:281 213:213 250:250 227:231 **162**:164 |
| 27 | **2** | 174:**178** 224:224 **258**:281 213:**220** 250:**252** 231:231 **162**:164 |
| 1 | **3** | 164:**178** 224:**226** 281:283 213:220 252:260 227:231 **162**:170 |
| 2 | **3** | 164:164 224:224 281:281 213:220 252:260 227:231 **166**:168 |
| 3 | **3** | 164:176 222:224 281:283 213:220 **250**:252 227:231 **162**:168 |
| 1 | **4** | **164**:172 224:224 281:281 213:220 250:252 227:**231** 162:162 |
| 2 | **4** | **170**:172 224:224 258:281 213:220 250:250 227:**231** 164:164 |
| 3 | **4** | 172:178 224:224 258:258 213:213 250:252 227:**231** 162:164 |
| 4 | **4** | 172:178 224:226 281:281 213:220 250:250 227:227 162:162 |
| 5 | **4** | 172:178 224:226 258:281 213:220 250:252 227:227 162:162 |
| 6 | **4** | **170**:178 224:224 258:258 213:220 250:252 227:227 162:**170** |
| 1 | **5** | **172**:176 224:226 281:281 213:220 250:252 227:**231** 162:**164** |
| 2 | **5** | 164:176 226:226 281:281 213:220 250:250 227:**231** 162:**164** |
| 3 | **5** | 164:176 224:224 281:281 213;220 252:**260** 227:**231** 162:**164** |
| 4 | **5** | 164:176 222:224 281:281 213:220 250:252 227:227 162:**164** |
| 5 | **5** | 164:164 226:**228** 281:281 213:213 250:**260** 227:**231** 162:**164** |
| 6 | **5** | 164:**172** 224:**228** 281:281 213:220 252:**260** 227:**231** 162:**164** |
| 7 | **5** | 164:176 224:226 281:281 213:213 250:252 227:227 162:**164** |
| 8 | **5** | 164:176 224:226 281:281 213:213 250:252 227:**231** 162:162 |
| 9 | **5** | **174**:176 224:226 281:281 213:220 250:252 227:**231** 162:162 |
| 10 | **5** | 164:176 226:226 281:281 213:220 250:250 227:**231** 162:**164** |
| 11 | **5** | 164:176 224:226 281:281 213:213 250:252 227:**231** **164**:162 |
| 12 | **5** | **172**:176 224:226 281:281 213:220 250:252 227:**231** 162:**164** |
| 1 | **6** | **172**:174 224:**225** 283:283 213:**221** 250:252 **227**:231 162:**164** |
| 2 | **6** | **164**:178 224:226 **281**:296 213:220 250:250 231:231 162:162 |
| 3 | **6** | **170**:178 224:226 296:296 213:220 250:252 **227**:231 162:**164** |
| 4 | **6** | **176**:178 224:224 **281**:296 213:220 252:**260** 231:**233** **164**:168 |
| 5 | **6** | **172**:174 224:224 296:296 213:220 250:252 **227**:231 **164**:168 |
| 6 | **6** | **164**:174 226:226 **281**:296 213:220 250:250 231:231 **164**:168 |
| 7 | **6** | **164**:174 224:**225** **281**:283 213:220 250:252 231:231 168:168 |
| 8 | **6** | **176**:178 224:226 **258**:296 213:220 250:250 231:231 162:168 |
| 9 | **6** | 174:178 224:226 **281**:296 213:220 250:252 231:231 162:**164** |
| 10 | **6** | **164**:174 224:224 **258**:296 213:220 250:252 **227**:231 162:162 |
| 11 | **6** | **172**:178 **222**:226 283:296 213:220 250:252 231:231 **164**:168 |
| 12 | **6** | **172**:174 224:224 283:283 213:**221** 250:252 **227**:231 162:**164** |
| 13 | **6** | **164**:178 226:226 283:296 213:220 250:250 231:231 **164**:168 |
| 14 | **6** | 174:178 224:226 283:296 213:**221**  250:252 231:231 **164**:168 |
| 15 | **6** | **164**:178 224:226 **281**:296 213:220 250:252 231:231 162:168 |
| 16 | **6** | 174:178 226:226 283:283 213:220 250:252 **227**:231 168:168 |
| 17 | **6** | 174:**176** 224:226 283:283 213:220 250:252 **227**:231 **164**:162 |
| 1 | **7** | **164**:174 226:226 281:281 213:220 250:250 231:231 **162**:164 |
| 2 | **7** | **164**:174 222:226 281:281 213;220 250:**252** 231:231 164:164 |
| 3 | **7** | 174:**178** 226:226 281:281 220:**221** 250:250 **227**:231 164:164 |
| 4 | **7** | **164**:174 226:226 281:281 213:220 250:250 231:231 164:164 |
| 5 | **7** | **164**:174 222:226 281:281 213:**221** 250:**252** 231:231 **162**:164 |
| 6 | **7** | 176:**178** 226:226 281:281 220:**221** 250:**252**  231:**256** 164:164 |
| 7 | **7** | **164**:174 222:226 281:281 213:**221** 250:250 231:**256** **162**:164 |
| 8 | **7** | 174:**178** 226:226 281:281 213:**221** 250:250 **227**:231 164:164 |
| 9 | **7** | **164**:174 222:226 281:281 213:220 250:**252** 231:**256** 164:164 |
| 1 | **8** | 164:176 224:224 281:281 213:**220** 252:252 231:231 164:164 |
| 2 | **8** | 164:164 224:226 281:281 213:221 250:252 231:231 **162**:164 |
| 3 | **8** | 164:176 224:226 281:281 213:**220** 250:252 231:231 164:164 |
| 4 | **8** | 164:176 224:224 281:281 213:**220** 250:252 231:231 **162**:164 |
| 5 | **8** | 164:**178** 224:226 281:281 213:**220** 250:252 227:231 162:164 |
